# Exploratory Study on the Associations between Lifetime Post-Traumatic Stress Spectrum, Sleep, and Circadian Rhythm Parameters in Patients with Bipolar Disorder

**DOI:** 10.3390/ijerph20043566

**Published:** 2023-02-17

**Authors:** Claudia Carmassi, Francy Cruz-Sanabria, Davide Gravina, Miriam Violi, Chiara Bonelli, Valerio Dell’Oste, Virginia Pedrinelli, Paolo Frumento, Ugo Faraguna, Liliana Dell’Osso

**Affiliations:** 1 Department of Clinical and Experimental Medicine, University of Pisa, 56126 Pisa, Italy; 2 Department of Translational Research and of New Surgical and Medical Technologies, University of Pisa, 56126 Pisa, Italy; 3 Department of Biotechnology, Chemistry and Pharmacy, University of Siena, 53100 Siena, Italy; 4 Department of Political Sciences, University of Pisa, 56126 Pisa, Italy; 5 Department of Developmental Neuroscience, IRCCS Stella Maris Foundation, 56128 Pisa, Italy

**Keywords:** chronotype, bipolar disorder, post-traumatic stress symptoms, rest–activity, sleep, actigraphy

## Abstract

The present study aimed at exploring whether lifetime post-traumatic stress spectrum symptoms are associated with chronotype in patients with bipolar disorder (BD). Moreover, we explored whether the chronotype can moderate the potential associations between lifetime post-traumatic stress spectrum symptoms and rest–activity circadian and sleep-related parameters. A total of 74 BD patients were administered the Trauma and Loss Spectrum Self-Report (TALS-SR) lifetime version for lifetime post-traumatic stress spectrum symptoms, the Pittsburgh Sleep Quality Index (PSQI) for self-reported sleep quality, and the Reduced Morningness–Eveningness Questionnaire (rMEQ) to discriminate evening chronotypes (ETs), neither chronotype (NT), and morning chronotype (MT). Actigraphic monitoring was used to objectively evaluate sleep and circadian parameters. Patients classified as ET reported significantly higher scores in the re-experiencing domain, as well as poorer sleep quality, lower sleep efficiency, increased wake after sleep onset, and delayed mid-sleep point compared with both NT and MT (*p*-value ≤ 0.05). Moreover, ET presented significantly higher scores in the TALS-SR maladaptive coping domain than NT and lower relative amplitude than MT (*p*-value ≤ 0.05). Moreover, higher TALS-SR total symptomatic domains scores were significantly correlated with poor self-reported sleep quality. Regression analyses showed that the PSQI score maintained the association with the TALS total symptomatic domains scores after adjusting for potentially confounding factors (age and sex) and that no interaction effect was observed between the chronotype and the PSQI. Conclusions: This exploratory study suggests that patients with BD classified as ET showed significantly higher lifetime post-traumatic stress spectrum symptoms and more disrupted sleep and circadian rhythmicity with respect to other chronotypes. Moreover, poorer self-reported sleep quality was significantly associated with lifetime post-traumatic stress spectrum symptoms. Further studies are required to confirm our results and to evaluate whether targeting sleep disturbances and eveningness can mitigate post-traumatic stress symptoms in BD.

## 1. Introduction

Sleep and circadian rhythm disturbances are frequently reported in bipolar disorder (BD) during both active episodes and euthymic phase [1,2,3], and have been associated with worse clinical outcomes such as interepisode dysfunction, adverse health outcomes, relapse [4], and reduced treatment response [5]. Moreover, the evening chronotype, which refers to the preference for later sleep and wake timings [6], is highly prevalent in BD and has been associated with worse clinical outcomes, including more relapses, more severe depressive episodes, and more frequent suicide attempts [7,8,9,10]. Furthermore, several studies have demonstrated that evening chronotype is associated with disturbed sleep also in healthy control subjects (HC) [11] and in patients with BD, in which eveningness has been correlated with insomnia [12] and increased sleep onset latency [13], as revealed by self-reported assessments. Nevertheless, so far reports in the literature evaluating whether sleep disturbances objectively measured are associated with chronotype in BD are scarce. Only one study investigated relationships between subjective self-report-based versus objective actigraphy-based chronotype in BD, finding that both assessments were significantly correlated with one-another and confirming prior associations between BD and evening chronotype [14]. To the best of our knowledge, no study has evaluated whether chronotype and sleep/circadian disturbances are associated with worse clinical outcomes in BD.

Another factor associated with worse clinical outcomes in BD is the comorbidity with other psychiatric disorders [15]. There is strong evidence of the link between BD and post-traumatic stress disorder (PTSD): patients with BD tend to report a major propensity to be exposed to traumatic events compared with the general population [16,17], and trauma exposure is associated with worse clinical outcomes in BD, including more frequent mood fluctuations, psychotic episodes, substance abuse, and suicidal ideation [18,19]. However, a prospective study reported that only less than one third of patients with BD exposed to trauma develop full-blown PTSD [20] and epidemiological studies have reported that rates of PTSD diagnosis in samples of patients with BD may vary between 4 and 40% [19]. This subset of patients with BD and comorbid PTSD is characterized by more frequent and severe depressive and manic symptoms, psychotic and self-injurious symptoms, and high suicide rates compared with patients with BD without PTSD [19]. These findings highlight the relevance of identifying factors that may be associated with worse stress reactions potentially increasing the risk of developing trauma and stressor-related disorders in patients with BD.

Among the factors that may worsen the reaction to stressful events in BD, sleep and circadian disturbances are relevant candidates. In PTSD, in fact, sleep disturbance is a core clinical feature regardless of age, sex, and psychiatric comorbidity [21], and subjective sleep disruption occurring early after the trauma may predict PTSD development [22,23]. Therefore, sleep disturbances have been suggested to be involved in the mechanisms underlying the onset and maintenance of PTSD [24]. Moreover, disturbances in circadian variables such as the robustness and/or regularity of rest–activity rhythms have been associated with PTSD. Compared with HC, patients with PTSD diagnosis exhibited greater intradaily variability [25] and lower interdaily stability, relative amplitude, and sleep regularity [26]. Furthermore, the circadian typology also has been associated with post-traumatic stress symptoms (PTSSs). In particular, the evening chronotype (ET), has been related to increased PTSSs compared with morning chronotypes (MTs) in a sample of firefighters [27], and eveningness has been correlated with disturbed sleep and more frequent and intense nightmares in a sample of military veterans [28].

Hence, there is consistent evidence in the literature regarding the high prevalence of evening chronotype, sleep/circadian disturbances, and elevated rates of PTSS in patients with BD. Likewise, associations between PTSS and sleep/circadian parameters have repeatedly been established. However, to the best of our knowledge, there is no literature exploring the relationship between lifetime PTSS and sleep/circadian parameters in patients with BD. Therefore, the aim of the present study is to explore whether lifetime post-traumatic stress spectrum symptoms are associated with chronotype in patients with BD. Moreover, we explored whether the chronotype can moderate the potential associations between lifetime post-traumatic stress spectrum symptoms and rest–activity circadian and sleep-related parameters.

## 2. Materials and Methods

### 2.1. Participants

A sample of patients with a diagnosis of BD attending the outpatient unit of the Psychiatric Clinic of the University of Pisa (Italy) were consecutively enrolled for the present study. Inclusion criteria consisted in a Diagnostic and Statistical Manual of Mental Illnesses, Fifth Edition, (DSM-5) diagnosis of BD (I–II) assessed by the Structured Clinical Interview for DSM-5 (SCID-5) [29] in the euthymic phase clinically assessed and confirmed by a total score ≤ 7 in the Hamilton Depression Rating Scale [30] and a total score ≤ 6 in the Young Mania Rating Scale [31], and age range between 18 and 65 years. Exclusion criteria included a clinical history of neurological disease and changes in psychopharmacological therapy (dosages or molecules) in the four weeks preceding the recruitment. All eligible subjects were asked to provide written informed consent after receiving a complete description of the study, and granted the opportunity to ask questions before joining the study and during its entire duration. The study was conducted in accordance with the Helsinki Declaration and received the approval of the Ethics Committee of AOUP, Area Vasta Toscana Nord Ovest (code 14785/2019). Participants were evaluated though the Trauma and Loss Spectrum (TALS-SR), the Reduced Morningness–Eveningness Questionnaire (rMEQ), The Pittsburgh Sleep Quality Index (PSQI), Actigraphic Registration, and a questionnaire asking for socio-demographic, lifestyle, and clinical variables.

### 2.2. Instruments Description

#### 2.2.1. Socio-Demographic Data and Lifestyle

Socio-demographic information included age, sex, height, weight, occupation and years of education. Participants were specifically asked whether their occupation required night shifts. Health-related lifestyle variables included alcohol and tobacco consumption. Alcohol intake was quantified as alcohol units per week (an alcohol unit corresponds to a half-pint of beer, a glass of wine or a measure of spirit). Tobacco intake was quantified as the number of cigarettes smoked in a week.

#### 2.2.2. The Trauma and Loss Spectrum (TALS-SR)

The TALS-SR questionnaire includes 116 items exploring the lifetime experience of a range of loss and/or traumatic events and lifetime symptoms, behaviors, and personal characteristics that might represent manifestations and/or risk factors for the development of a stress-response syndrome [32]. Items responses are coded in a dichotomous way (yes/no). The instrument is organized into nine domains, investigating specifically: loss events (I); grief reactions (II); potentially traumatic events (III); reactions to losses or upsetting events (IV); re-experiencing (V); avoidance and numbing (VI); maladaptive coping (VII); arousal (VIII); and personal characteristics/risk factors (IX). The questionnaire investigates both a comprehensive set of traumatic and loss events that may occur in the lifetime and a broad spectrum of symptoms related to these events. In the present study, we calculated the TALS-SR symptomatic domain, which corresponds to the sum of the TALS-SR domains that account for the clinical symptoms of the post-traumatic spectrum (Domains: IV, V, VI, VII, and VIII).

#### 2.2.3. The Reduced Morningness–Eveningness Questionnaire (rMEQ)

The rMEQ is the reduced version of the Horne–Ostberg Morningness–Eveningness Questionnaire [33]. It describes the individual preference for daily rhythms and activities, as well as the timing of the individual sleep/wake patterns, allowing to identify the three chronotypes based on the total score: Morning Type (MT) = 19–25, Neither Type (NT) = 11–18, Evening Type (ET) = 4–10. The cut points were established following the recommendations derived from the validation of the rMEQ for the Italian population [34,35].

#### 2.2.4. The Pittsburgh Sleep Quality Index (PSQI)

The PSQI is a self-rated questionnaire that assesses sleep quality and disturbances over a 1-month time interval, providing scores for subjective sleep quality, sleep latency, sleep duration, habitual sleep efficiency, sleep disturbances, use of sleep medication, and daytime dysfunction [36]. The PSQI was translated and adapted into Italian by [37]. The sum of scores for the seven components yields one global score, which is interpreted as follows: Minimum Score = 0 (better); Maximum Score = 21 (worse). Score *>* 5: poor sleep quality.

#### 2.2.5. Actigraphic Registration

Each participant was equipped with a waterproof wrist actigraph, Fitbit Flex2 (FF2), recording accelerometric activity continuously from the non-dominant wrist for 7 days. Data were sampled in 1 min epochs and digitally stored for subsequent analysis.

The quantitative sleep parameters derived from the FF2 were estimated through an artificial neural network (ANN) based and certified algorithm (Dormi by sleepActa s.r.l.), validated in a sample including both healthy subjects and patients undergoing a diagnostic exam for sleep disturbances [38,39]. The Dormi software is a medical, risk class I device. As such, it is registered within the Italian Ministry of Health Data Bank of Medical Devices (CND: 217 Z12030682). The parameters estimated were:

- Total sleep time (TST): the total amount of time spent asleep. Calculation: sum of all asleep time within a sleep period (in hours). Wakefulness duration after initial sleep onset (WASO): the amount of time spent awake during a sleep period. Calculation: the amount of time spent awake from initial sleep onset until final awakening (in minutes). Sleep efficiency (SE): total sleep time as a percentage of the total amount of time there was an intention to sleep. Calculation: TST/Time when individual is attempting to sleep (TATS)*100. Based on the technical publication of Definitions and Characteristics for Wearable Sleep Monitors [40].
- Sleep regularity index: measures the likelihood of the same sleep–wake state occurring in epochs that are 24 h apart, thereby measuring the similarity of sleep–wake patterns between consecutive days [41].
- Mid-sleep point: the middle of the sleep period between the sleep onset and final awakening, measured by actigraphy. Calculated by adding to the average sleep onset half of the average total sleep time (average sleep onset + average TST/2).

To estimate rest–activity circadian rhythm parameters, we used a parametric approach using the cosinor method to characterize diurnal rhythms by fitting a sine wave to the actigraphy data and computing the Midline Estimating Statistic of Rhythm (MESOR), amplitude, and acrophase [42]. To perform the cosinor analysis we used the R package Extract Circadian Rhythms Metrics from Actigraphy Data ‘ActCR’ [43]. Non-parametric measurements of rest–activity rhythms, such as interdaily stability, intradaily variability, and relative amplitude [44] were calculated using the R package Non-Parametric Measures of Actigraphy Data ‘nparACT’ [45,46]. Variables are defined as follows:

- Mesor: a rhythm-adjusted mean. It represents the mean activity level;
- Amplitude: the peak-to-nadir difference, a measure of half the extent of predictable variation within a cycle. More robust rhythms have a higher amplitude;
- Acrophase: timing of peak activity or the point in the cycle with highest activity;
- Interdaily stability (IS): estimates the variability in rest–activity patterns across all days. It is a measure of rest–activity rhythms regularity. In this study, it is expressed as values ranging from 0 to 1. Higher values indicate greater stability between days;
- Intradaily variability (IV): quantifies the fragmentation and magnitude of rest–activity transitions within each day. In this study, it is expressed as values ranging from 0 to approximately 2. Higher values indicate frequent transitions between rest and activity (i.e., frequent naps, increased night-time awakenings);
- Relative amplitude: measures the robustness of the 24 h rest–activity rhythm by calculating the normalized mean difference in activity between the most active 10 h and the least active 5 h, ranging from 0 to 1. Higher values indicate lower activity during the night and high activity during the day, i.e., increased robustness of rest–activity rhythm.

### 2.3. Statistical Analyses

Data distribution was evaluated through the Shapiro–Wilk test finding that most of the analyzed variables showed a *p*-value < 0.05 (suggesting that data distribution was significantly different from normal distribution), see Table S1 of Supplementary Material. Moreover, the sample size of chronotype subgroups (ET and MT) was *n* < 30. Therefore, we used non-parametric tests for comparison between chronotypes and for correlation analyses. Comparisons between groups were performed using the Kruskal–Wallis and post-hoc pairwise Mann–Whitney–Wilcoxon rank-sum test for continuous variables and the Fisher exact test for categorical variables. The Spearman’s correlation test was performed to evaluate whether sleep-related and circadian parameters can be correlated to higher scores in the TALS-SR symptomatic domains. Holm correction for multiple comparisons was performed for post-hoc and correlations analyses. Significant results were considered at *p*-value ≤ 0.05. Linear regression models were estimated to explore the association between the TALS-SR symptomatic domains and sleep/circadian parameters that were found to be significantly correlated to the TALS-SR symptomatic domains, adjusting for potential confounding factors (e.g., age and sex). Then, regression analyses were performed to explore the role of chronotype as a moderator of the associations between sleep/circadian parameters and TALS symptomatic domains. All statistical analyses were conducted using R Studio 4.1.3 version.

## 3. Results

### 3.1. Sample Description

**Figure 1:**
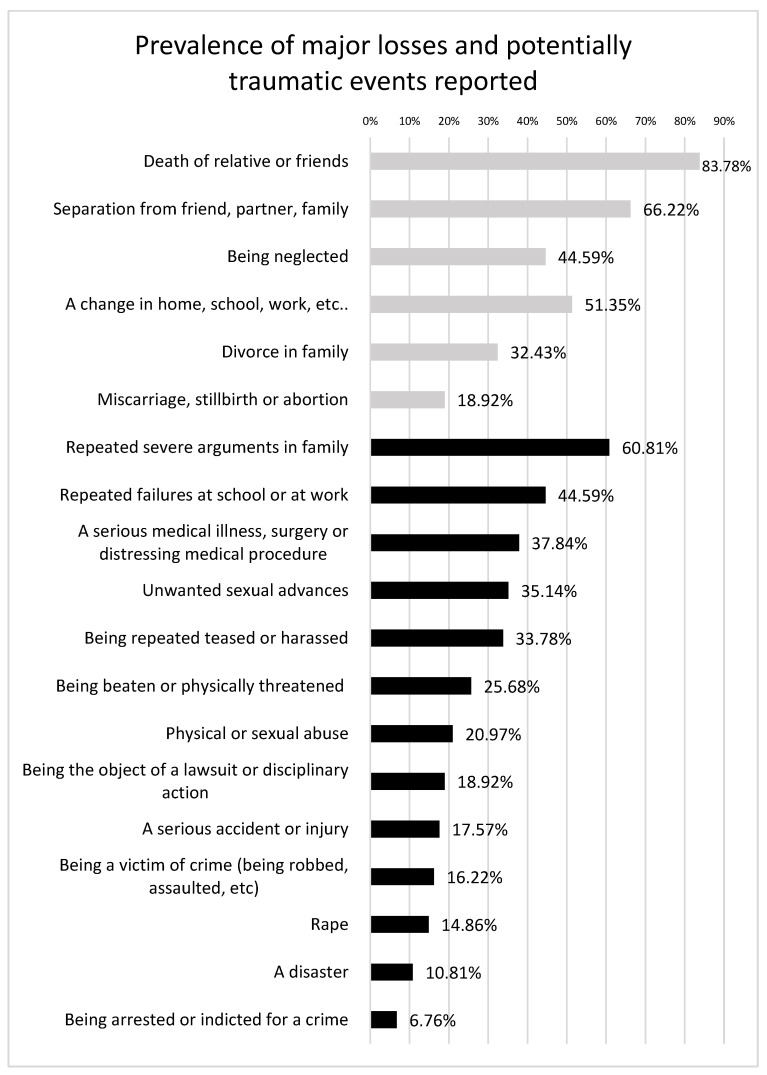
Prevalence of major losses (depicted in grey) and potentially traumatic events (depicted in black) reported.

Socio-demographic characteristics of the sample (*n* = 74, age 45 ± 13.09, males 54%) are presented in Table 1. The prevalence of major losses and potentially traumatic events reported are depicted in Figure 1.

Overall, in our sample each subject has experienced around 4.80 ± 2.002 loss events throughout his/her life, while only one subject from the total sample (1.35%) reported no loss event. The loss event more frequently reported was the death of a relative or loved friends (83.78%), the loss or death, separation from a close friend romantic partner, or family member because of relocation, hospitalization, military service, or because of an argument or disagreement (66.22%), a change in home, caregivers, schools, jobs, etc. (51.35%), being neglected or abandoned (44.59%), divorce in family (32.43%), a miscarriage, stillbirth, or abortion (18.92%). Overall, in our sample each subject has experienced around 3.91 ± 3.07 potentially traumatic events in their lifetime, while five (6.75%) subjects reported not having experienced a potentially traumatic event. Among the potentially traumatic events reported, the event more frequently reported was repeated severe arguments in family (60.81%), followed by repeated failures in school or at work (44.59%), having a serious medical illness, surgery, or other distressing medical procedure (37.84%). receiving unwanted sexual advances (35.14%), being repeatedly teased or harassed (33.78%), being beaten up or physically threatened (25.68%), and physical or sexual abuse (20.97%). Furthermore, some participants reported being the object of a lawsuit or disciplinary action (18.92%); experiencing a serious accident or injury (17.57%); being a victim of a crime such as being robbed, assaulted, etc. (16.22%); experiencing rape (14.86%); experiencing a natural disaster (10.81%); and being arrested or indicted for a crime (6.76%). No patient reported not having experienced either of the two types of events (potentially traumatic events and loss events), so all patients compiled TALS-SR symptomatic domains.

### 3.2. Comparisons between Chronotypes

The sample was divided into three circadian typologies as measured through the rMEQ: Evening Type (ET, *n* = 27 (36%)), Neither Type (NT, *n* = 33 (45.5%)), and Morning Type (MT, *n* = 14 (18.9%)). No differences between groups emerged in demographic and lifestyle variables. Regarding the TALS-SR domains, significant differences emerged between groups in several TALS-SR domains. Post-hoc analyses (adjusted *p*-values reported in Table S2 of Supplementary Material) showed that subjects classified as ET reported significantly higher scores than both NT and MT in the TALS-SR re-experiencing domain. Moreover, ET presented higher scores than NT in the TALS-SR maladaptive coping domain. With respect to sleep parameters, differences between groups emerged in the PSQI total score, Actigraphic metrics of SE, WASO, SRI, acrophase, mesor, interdaily stability, relative amplitude, and mid-sleep point. Post-hoc analyses revealed that ET reported poorer sleep quality, and showed lower SE, increased WASO, and delayed mid-sleep point than NT and MT, IS and SRI were also lower in ET compared with MT, while the MT showed an advanced acrophase and higher relative amplitude than NT and ET. Finally, the MT group showed lower mesor than the NT group (Table 1).

### 3.3. Correlations between TALS-SR Symptomatic Domains and Circadian and Sleep-Related Parameters

Correlations analyses adjusted for multiple comparisons showed that the PSQI total score was significantly and positively correlated with the TALS-SR symptomatic domains (rho = 0.46, unadjusted *p*-value =< 0.001, and adjusted *p*-value = 0.005), in which higher scores in the PSQI, suggestive of poor sleep quality, correlated with more severe stress reactions. Although the TALS-SR symptomatic domains were also correlated with the rMEQ total score (rho = −0.25, unadjusted *p*-value = 0.031, and adjusted *p*-value = 0.341), the intradaily variability (rho = 0.34, unadjusted *p*-value = 0.005, and adjusted *p*-value = 0.538), and the interdaily stability (rho = 0.023, unadjusted *p*-value = 0.050, and adjusted *p*-value > 0.999), these correlations did not remain significant after correction for multiple comparisons (See Table 2 and Table 3).

### 3.4. Associations between Sleep/Circadian Parameters and TALS-SR Symptomatic Domains Adjusting for Potentially Confounding Factors and Chronotype

Previously we found that the PSQI was significantly correlated with TALS-SR symptomatic domains. Therefore, we estimated linear regression models using the TALS-SR symptomatic domains as the dependent variable and introduced the PSQI as the independent variable adjusting for potentially confounding factors (age and sex). This regression analyses showed that the PSQI remained associated with TALS symptomatic domains (β = 1.442, *p*-value =< 0.001), see Table 4. The residuals obtained in this model passed the normality test (Shapiro–Wilk *p*-value = 0.2939).

Finally, the chronotype was added to the model to explore its potential effect as a moderator of the associations between sleep/circadian and TALS symptomatic domains. We found the PSQI remained significantly associated with TALS symptomatic domains, where higher scores in the PSQI (suggestive of poorer seep quality) were associated with higher scores in the TALS symptomatic domains (β = 1.32, *p*-value = <0.011); however, no interaction effect with chronotype was found. (PSQI * Chronotype (ET vs. NT) β = 0.435, *p*-value = 0.203 and PSQI * Chronotype (ET vs. MT) β = −1.125, *p*-value = 0.271); see Table 5. The residuals obtained in this model passed the normality test (Shapiro–Wilk *p*-value = 0.2723). Goodness-of-fit measures (adjusted R^2^) suggest that the model including only the subjective sleep quality, as measured through the PSQI, was associated with PTSS to a greater extent than the model including the chronotype as a potential moderator (adjusted R^2^ = 0.28 vs. adjusted R^2^ = 0.26, respectively). The variance inflation factor (VIF) and tolerance values of the model in which we estimated simultaneously the effect of the chronotype and the PSQI were calculated. We found that VIF values were <10 and tolerance values were higher than 0.1, suggesting that multicollinearity was not significant, see Table S3 of supplementary material.

## 4. Discussion

In the present study, for the first time we report the association between ET and higher lifetime post-traumatic stress spectrum symptoms, more disrupted sleep and circadian rhythmicity with respect to MT and NT, among a sample of patients with BD. Our results are consistent with previous findings showing how eveningness was associated with higher PTSS in subjects exposed to potentially traumatic events [27,47]. Likewise, associations between eveningness and symptoms that are related to PTSS have been reported, such as increased tendency to maladaptive behaviors [48,49], propensity to experience nightmares [50,51], and lower levels of resilience to stressful events [52]. Our results extend previous findings reported in the literature by showing that among BD patients, those classified as ET tend to report more severe lifetime post-traumatic stress spectrum symptoms, particularly more severe re-experiencing and maladaptive coping symptoms compared with NT and MT, thus suggesting that BD patients classified as ET can represent a subset of patients with an increased risk to develop stress-related disorders. Interestingly, our findings also showed more, despite not being statistically significant, lifetime losses and potentially traumatic events among ET patients with BD than NT and MT. Recently, some of us found that eveningness is correlated with higher lifetime post-traumatic stress spectrum symptoms in HC [53] suggesting a vulnerability to worse stress reactions among subjects with evening preference even when not reporting a diagnosis of bipolar disorder.

Several studies have reported the propensity of ET to experience sleep disturbances, measured subjectively and objectively [11,54]. Accordingly, in patients with BD, previous studies using self-report instruments have reported that eveningness is correlated with insomnia [12] and with augmented sleep onset latency [13]. Moreover, Gershon et al., 2018, found that BD patients reported more eveningness than HC and that subjective and objective assessments of chronotypes were significantly correlated [14]. Accordingly, our results showed that the mid-sleep point and the acrophase were associated with the self-reported chronotype. In addition, we also found that patients with BD classified as ET compared with NT and MT showed self-reported poorer sleep quality, disturbed sleep objectively measured as indicated by lower sleep efficiency, and increased wake after sleep onset. Moreover, interdaily stability and sleep regularity were lower in ET compared with MT, indicating reduced stability in the sleep–wake patterns along days in the ET group. Other authors have highlighted the link between delayed sleep phase and irregular sleep patterns [41,55], and between sleep irregularity and outcomes in mental and physical health [56,57]. Our results, therefore, highlight that eveningness may be associated with subjective and objective sleep and circadian disturbances in BD.

The interaction between eveningness, sleep disturbances, and variables related with worse clinical outcomes such as more severe stress reactions have not been explored before in BD. However, the role of sleep disturbances and comorbid PTSD and BD has begun to be explored. A recent study emphasized the observation that subjects with severe mental illnesses, including BD, and PTSD, have greater sleep disorders than those with only PTSD diagnosis [58]. The associations between PTSD symptoms and sleep disturbances have been reported in both patients with PTSD [59,60,61] and in cases of no full-blown diagnosis of PTSD [62,63]. We observed correlations between lifetime post-traumatic stress spectrum symptoms (TALS symptomatic domains) and sleep-related variables (self-reported sleep quality, sleep efficiency, and total sleep time) and circadian rhythm parameters (intradaily variability and interdaily stability) in our sample of patients diagnosed with BD. Among these, only the sleep quality remained significantly correlated with the TALS-SR symptomatic domains after the correction for multiple comparisons. Moreover, regression models showed that the self-reported sleep quality remained significantly associated with the TALS-SR symptomatic domains score, even after adjusting for age, sex, and chronotype. Previous studies have noticed that the PSQI is associated with mental health outcomes, even stronger than with objective measurements of sleep [64], thus highlighting the intimate association between the perceived sleep quality and mental health. However, our results are exploratory and must be replicated in larger samples that would allow for better control of the effect of potentially confounding factors, such as age and sex, and that allows to have a greater representativeness of each chronotype in order to better evaluate the potential role of a chronotype as a moderator of the associations between PTSS and sleep/circadian disturbances.

Other limitations, besides the sample size, must be taken into account when discussing our results. In particular, our study is cross-sectional and a causal link between trauma exposure and sleep disturbances cannot be established. Furthermore, a heterogeneous type of potentially traumatic events was evaluated thus including subjects exposed to events of several stress magnitudes. Indeed, some of our patients (*n* = 27, 36.48%) fulfilled criteria for PTSD assessed by means of TALS-SR items endorsed corresponding to DSM-5 criteria for PTSD diagnosis as previously described [19,65]. Although we found that the prevalence of PTSD diagnosis was not different between chronotype groups, further studies with larger sample size can explore the associations between chronotype and PTSS differentiating by patients with BD with comorbid PTSD, compared with BD patients without it. Moreover, although the pharmacological treatment can be a confounder factor of our results, comparisons between chronotype groups regarding clinical variables showed that groups only significantly differ regarding the disease onset, being younger in ET than NT and MT. Instead, no differences emerged with respect to the pharmacological treatment, nor regarding other clinical variables (see Table S4 of Supplementary Material). Finally, another limitation of our study is that we performed multiple tests that would increase the possibility of false positives by which the level of significance was adjusted. Some authors have suggested that data from exploratory studies should be analyzed without multiplicity adjustment, reporting ‘significant’ results as ‘exploratory results’ that should lead to hypotheses that can be tested in subsequent confirmatory studies [66]. Therefore, we reported also the correlations that were significant using unadjusted *p*-values to indicate potential associations that can guide further confirmatory studies in the field, based on these results further studies can explore the possible correlations between PTSS and chronotype, interdaily stability, and intradaily variability in patients with BD.

Despite the limitations described, our study remains the first to explore lifetime post-traumatic stress spectrum symptoms and sleep and circadian rhythm parameters in a sample of patients with BD, concluding that patients with BD and evening chronotype showed higher lifetime post-traumatic stress spectrum symptoms and more disrupted sleep and circadian rhythmicity than neither and morning chronotypes.

Mechanisms underlying the associations between PTSS and sleep/circadian parameters in patients with BD remain to be elucidated; however, some hypotheses can be considered. On the one hand, there is increasing evidence about the strong association between mood disturbances and PTSS. It has been proposed that the impulsivity and excessive optimism that occur during hypomanic or manic episodes can lead patients to engage in potentially traumatic activities [67,68] and that cyclothymic mood reactivity and emotional instability may favor the development of PTSD in appropriate circumstances [69]. In particular, the hyperexcitation characteristic of mania, hypomania, or mixed states can have an impact on the symptoms experienced at the time of trauma (degree of fear of death and loss of control), thus being etiologically relevant for the emergency and maintenance of PTSD [68]. Moreover, subthreshold mood symptoms have been associated with an increased vulnerability to PTSS, especially in male subjects [67,69], in healthy subjects with autistic traits [70], and in subjects who experience work-related stress [71,72]. Likewise, subthreshold manic/hypomanic symptoms have been correlated with worse clinical outcomes, such as increased suicidal ideation, in patients with PTSD [73].

On the other hand, sleep and circadian disturbances may contribute to PTSS. In particular, eveningness can be associated with an increased exposure to potentially traumatic events and with worse stress reactions, so that in subjects classified as ET have been reported an increased propensity to risk behaviors [74] and greater maladaptive behaviors [48,49] compared with other chronotypes. Moreover, circadian misalignment derived from disturbed sleep–wake patterns can predispose to reward system alterations [75], thus possibly explaining the increasing reward seeking that has been reported in ET. Likewise, sleep disturbances have been associated with decreased inhibitory control, impulsivity, and risk propensity [76], possibly contributing to the likeliness to be engaged in risk behaviors that may represent potentially traumatic events. In this way, in patients with BD, factors such as the evening chronotype and disrupted sleep/circadian patterns can interact, synergistically contributing to an increased propensity to be exposed to situations that may represent a threat and that constitute potentially traumatic events, and this risk may be significantly increased during manic or hypomanic episodes due to the increased goal-directed behaviors characteristic of these episodes. In a recent study, it was seen how the interpersonal psychotherapy and social rhythm therapy (IPSRT), focused on the reorganization of social rhythms and the increase in skills to cope with social stressors, can be effective in improving the clinical symptomology of BD patients [77]. Likewise, despite the widespread belief that chronotype is an unmodifiable trait, several studies have demonstrated successful interventions in advancing circadian preference through manipulating light exposure [78] or by using a Transdiagnostic Sleep and Circadian Intervention [79] in HC. Similarly, in patients with a major depressive disorder, a move towards morningness has been observed after an antidepressant treatment based on agomelatine [80] and after a chronotherapy based on bright light in which the change in evening preference predicted a greater probability of remission of depression over 5 months of follow-up [81]. Although other authors have found that the chronotype remained stable from admission to discharge in a sample of patients with nonseasonal depressive disorder managed with heterogeneous treatments [82], the circadian preference remains a relevant intervention target in patients with mood disorders. In this context, the chronobiotic effect of exogenous melatonin [83] represents a promising alternative for the regularization of sleep/wake patterns in patients with BD. Our results suggest that targeting sleep disturbances and regularizing circadian rhythms can be particularly useful in patients with BD classified as an evening chronotype, in which these interventions might allow to modify clinical outcomes such as the propensity to risk behaviors, as well as the exposure and reaction to potentially traumatic events.

## 5. Conclusions

Our results suggest that in patients with bipolar disorder classified as evening chronotype, sleep and circadian disturbances may be more pronounced than in the other chronotypes, and simultaneously this subset of patients (evening chronotypes) tend to experience worse lifetime post-traumatic stress spectrum symptoms, being possibly at a higher risk to develop stress-related disorders. Considering the evidence about the interaction between post-traumatic stress symptoms and sleep/circadian parameters, further studies are required to evaluate whether interventions targeting sleep and circadian disturbances may mitigate post-traumatic stress symptoms in bipolar disorder.

## Figures and Tables

**Table 1:** Descriptive data and comparisons between chronotype groups (defined through the rMEQ) among patients with BD.

	Evening Type	Neither Type	Morning Type		
	*n* = 27	*n* = 33	*n* = 14	*p*-Value	Post-Hoc
Age, years	48.00 [34.50, 54.50]	48.00 [38.00, 57.00]	47.50 [39.25, 55.75]	0.825	
Sex, male (%)	12 (44%)	19 (57.6%)	9 (63.3%)	0.420	
Education, years	13.00 [13.00, 17.00]	13.00 [13.00, 17.00]	13.00 [13.00, 19.00]	0.858	
Alcohol intake, u/w	0.00 [0.00, 1.00]	0.00 [0.00, 1.00]	0.00 [0.00, 0.75]	0.889	
Tobacco intake, u/w	0.00 [0.00, 56.00]	0.00 [0.00, 23.75]	0.00 [0.00, 0.00]	0.605	
BMI, kg/m^2^	25.16 [20.39, 28.77]	25.81 [23.55, 28.92]	27.71 [24.85, 30.66]	0.191	
TALS-SR Loss events	6.00 [4.00, 7.00]	4.00 [4.00, 6.00]	4.50 [3.00, 5.75]	0.062	
TALS-SR Grief reactions	14.00 [8.50, 17.50]	11.00 [8.00, 13.00]	11.50 [6.75, 12.75]	0.151	
TALS-SR Potentially traumatic events	7.00 [3.00, 9.00]	4.00 [2.00, 6.00]	3.50 [2.00, 6.75]	0.070	
TALS-SR Reaction to losses and PTE	10.00 [5.00, 14.50]	6.00 [2.00, 11.00]	6.00 [3.25, 8.00]	0.037 *	ns
TALS-SR Re-experiencing	5.00 [3.50, 8.00]	2.00 [0.00, 5.00]	3.00 [1.00, 4.00]	0.005 *	a,b
TALS-SR Avoidance and numbing	5.00 [4.00, 9.00]	4.00 [0.00, 8.00]	4.00 [1.25, 6.75]	0.154	
TALS-SR Maladaptive coping	3.00 [0.00, 4.50]	0.00 [0.00, 3.00]	0.50 [0.00, 1.00]	0.043 *	b
TALS-SR Arousal	3.00 [1.00, 4.00]	2.00 [1.00, 4.00]	2.50 [0.25, 4.00]	0.545	
TALS-SR Symptomatic domains	27.00 [14.50, 39.00]	16.00 [8.00, 27.00]	17.50 [7.50, 24.50]	0.038 *	ns
PSQI Total score	12.00 [9.00, 15.00]	7.00 [5.00, 12.00]	8.00 [4.50, 9.00]	0.001 *	a,b
SE, %	88.00 [81.90, 91.50]	90.50 [85.85, 96.90]	93.15 [90.33, 95.82]	0.010 *	a,b
WASO, minutes	58.00 [40.50, 94.50]	45.50 [16.25, 56.50]	33.00 [26.00, 42.75]	0.013 *	a,b
TST, hour	7.20 [6.25, 8.80]	7.50 [6.80, 8.10]	8.55 [7.28, 9.47]	0.096	
SRI	61.30 [54.05, 73.70]	71.35 [56.25, 81.92]	76.50 [70.83, 81.12]	0.014 *	b
Acrophase, hh:mm	16:34 [15:58, 17:24]	15:54 [15:14, 16:54]	14:52 [14:34, 15:28]	<0.001 *	b,c
Amplitude	0.37 [0.34, 0.42]	0.38 [0.34, 0.42]	0.40 [0.37, 0.46]	0.195	
Mesor	0.58 [0.54, 0.64]	0.60 [0.55, 0.65]	0.53 [0.50, 0.57]	0.034 *	c
Interdaily stability	0.78 [0.70, 0.82]	0.82 [0.74, 0.87]	0.86 [0.82, 0.92]	0.020 *	b
Intradaily variability	0.33 [0.28, 0.40]	0.32 [0.28, 0.42]	0.34 [0.30, 0.39]	0.889	
Relative amplitude	0.76 [0.62, 0.84]	0.78 [0.69, 0.83]	0.88 [0.84, 0.91]	0.004 *	b,c
Mid-sleep point, hour	4.65 [4.20, 5.16]	3.94 [3.31, 4.35]	2.33 [2.02, 2.80]	<0.001 *	a,b,c

Abbreviations: BMI: Body Mass Index; Alcohol intake units per week (u/w): Alcohol units per week; Tobacco intake u/w: Tobacco (cigarettes) units per week; TALS-SR: Trauma and Loss Spectrum Lifetime version; PTEs: potentially traumatic events; PSQI: Pittsburgh Sleep Quality Index total score; SE: sleep efficiency; WASO: Wake After Sleep Onset; TST: total sleep time; ns: non-significant. Acrophase is reported as hours and minutes (hh:mm). TST and Mid-sleep point are reported as fractions of hours. Results are presented in median (IQR: Interquartile range) for continuous variables and as frequency and percentage for categorical ones. Comparisons between groups were performed through the Kruskal–Wallis for continuous variables and the Fisher test for categorical variables. Post-hoc pairwise Mann–Whitney–Wilcoxon rank-sum test adjusted *p*-values are reported in Supplementary Material Table S2. Significant results are reported here as follows: a Significant difference between Evening Type and Neither Type groups. b Significant difference between Evening type and Morning Type groups. c Significant difference between Neither Type and Morning Type groups. (*) Significant values were considered at *p*-value ≤ 0.05 (adjusted).

**Table 2:** Correlation values between TALS-SR domains and subjective and objective sleep parameters.

	PSQI	WASO	TST	SE	SRI
	rho	rho	rho	rho	rho
	(*p*-Value)	(*p*-Value)	(*p*-Value)	(*p*-Value)	(*p*-Value)
TALS-SR Symptomatic domains	0.46	0.20	0.04	−0.22	0.01
	0.005 *	0.751	>0.999	0.543	>0.999

Abbreviations: TALS-SR: Trauma and Loss Spectrum Lifetime version; PSQI: Pittsburgh Sleep Quality Index; SE: sleep efficiency; WASO: Wake After Sleep Onset; TST: total sleep time. Results are presented as Spearman’s rank correlation coefficient (rho) and *p*-values with Holm correction for multiple comparisons. (*) Significant correlations were considered at *p*-value ≤ 0.05.

**Table 3:** Correlation values between TALS-SR domains and subjective and objective circadian parameters.

	rMEQ	Mesor	Amplitude	Acrophase	IV	IS	RA	Mid-Sleep Point
	rho	rho	rho	rho	rho	rho	rho	rho
	(*p*-Value)	(*p*-Value)	(*p*-Value)	(*p*-Value)	(*p*-Value)	(*p*-Value)	(*p*-Value)	(*p*-Value)
TALS-SR Symptomatic domains	−0.25	−0.06	−0.17	−0.17	0.34	−0.23	0.07	−0.11
	0.341	>0.999	>0.999	0.061	0.538	>0.999	>0.999	>0.999

Abbreviations: TALS-SR: Trauma and Loss Spectrum Lifetime version; rMEQ: Morningness–Eveningness Questionnaire reduced version; IS: Interdaily stability; IV: Intradaily variability; RA: Relative amplitude. Results are presented as Spearman’s rank correlation coefficient (rho) and *p*-values with Holm correction for multiple comparisons. Significant correlations were considered at *p*-value ≤ 0.05.

**Table 4:** Regression analysis estimating the associations between sleep/circadian parameters and TALS-SR symptomatic domains.

Outcome: TALS Symptomatic Domains			
	β	*p*-Value	95% CI
Age	−0.294	0.005 *	−0.50 −0.08
Sex (male)	0.008	0.997 *	−4.45 5.47
PSQI	1.442	<0.001 *	0.85 2.03
R^2^	0.28		

The TALS-SR symptomatic domain corresponds to the sum of the TALS-SR domains that account for the clinical symptoms of the post-traumatic spectrum: Domain IV Reaction to losses and potentially traumatic events, Domain V Re-experiencing, Domain VI Avoidance and numbing, Domain VII Maladaptive coping, and Domain VIII Arousal. The model was adjusted for age and sex. (*) Significant values were considered at *p*-value ≤ 0.05.

**Table 5:** Regression analyses estimating the associations between sleep quality and TALS-SR symptomatic domains evaluating the potential effect of chronotype as a moderator.

Outcome: TALS Symptomatic Domains			
	β	*p*-Value	95% CI
Age	−0.293	0.007	−0.50 −0.08
Sex (male)	−0.258	0.926	−5.28 5.80
PSQI	1.320	0.011 *	0.30 2.33
Chronotype (ET vs. NT)	−5.573	0.481	−21.28 10.13
Chronotype (ET vs. MT)	6.154	0.533	−13.48 25.79
global *p*-value of Chronotype	0.797		
PSQI * Chronotype (ET vs. NT)	0.435	0.203	−0.99 1.86
PSQI * Chronotype (ET vs. MT)	−1.125	0.271	−3.15 0.90
global *p*-value of interaction	0.312		
R^2^	0.26		

The TALS-SR symptomatic domain corresponds to the sum of the TALS-SR domains that account for the clinical symptoms of the post-traumatic spectrum: Domain IV Reaction to losses and potentially traumatic events, Domain V Re-experiencing, Domain VI Avoidance and numbing, Domain VII Maladaptive coping, and Domain VIII Arousal. Model was adjusted for age and sex. (*) Significant values were considered at *p*-value ≤ 0.05.

## Data Availability

All data generated or analyzed during this study are included in the manuscript.

## Supplementary Materials

The following supporting information can be downloaded at: https://www.mdpi.com/article/10.3390/ijerph20043566/s1, Table S1: Normal distribution test; Table S2: Post-hoc analyses *p*-values; Table S3: Multicollinearity test. Table S4: Comparison between chronotypes regarding clinical variables.
