# Supplementary material for: Exploratory Study on the Associations between Lifetime Post-Traumatic Stress Spectrum, Sleep, and Circadian Rhythm Parameters in Patients with Bipolar Disorder
Source: Int J Environ Res Public Health. 2023 Feb 17;20(4):3566. doi: 10.3390/ijerph20043566 (PMC9967425; doi:10.3390/ijerph20043566)
Supplement: Supplementary file 1 [file ijerph-20-03566-s001.zip › ijerph-2122578-supplementary.pdf]

Table S1. Normal distribution test

	Overall sample (n=74)		Normality test
	(mean (SD))	(median [IQR])	p-value
Age, years	45.43 (13.09)	48.00 [36.00, 56.00]	0.007*
Education, years	14.55 (3.51)	13.00 [13.00, 17.00]	<0.001*
Alcohol intake, u/w	2.02 (5.29)	0.00 [0.00, 1.00]	<0.001*
Tobacco intake, u/w	33.58 (68.53)	0.00 [0.00, 35.00]	<0.001*
BMI, kg/m^2	26.77 (5.62)	26.15 [23.55, 29.62]	0.032*
TALS-SR Loss events	4.91 (1.97)	5.00 [4.00, 6.00]	0.080
TALS-SR Grief reactions	11.92 (5.96)	12.00 [8.00, 15.00]	0.262
TALS-SR Potentially traumatic events	5.12 (3.29)	4.50 [2.25, 7.00]	0.001*
TALS-SR Reaction to losses and PTE	7.49 (4.90)	7.00 [4.00, 11.75]	0.020*
TALS-SR Re-experiencing	3.70 (2.90)	3.00 [1.00, 6.00]	<0.001*
TALS-SR Avoidance and numbing	4.86 (3.54)	5.00 [1.25, 8.00]	<0.001*
TALS-SR Maladaptive coping	2.07 (2.42)	1.00 [0.00, 3.75]	<0.001*
TALS-SR Arousal	2.49 (1.78)	3.00 [1.00, 4.00]	<0.001*
TALS-SR Symptomatic domains	20.39 (13.67)	19.00 [9.00, 30.75]	0.006*
MEQ total score	12.78 (5.04)	12.00 [8.00, 17.00]	0.019*
PSQI Total score	9.28 (4.63)	9.00 [6.00, 13.00]	0.127
SE, %	88.45 (8.98)	90.00 [85.40, 94.00]	<0.001*
WASO, minutes	53.08 (40.14)	44.00 [26.00, 64.00]	<0.001*
TST, hour	7.77 (1.62)	7.50 [6.80, 8.60]	0.063
SRI	66.70 (16.25)	70.30 [56.77, 79.78]	<0.001*
Acrophase, hh:mm	974.39 (97.26)	966.00 [914.50, 1018.50]	<0.001*
Amplitude	0.38 (0.07)	0.38 [0.34, 0.42]	<0.001*
Mesor	0.58 (0.08)	0.58 [0.53, 0.63]	0.407
Interdaily stability	0.79 (0.11)	0.81 [0.73, 0.88]	<0.001*
Intradaily variability	0.35 (0.10)	0.33 [0.28, 0.41]	<0.001*
Relative amplitude	0.76 (0.14)	0.78 [0.69, 0.88]	0.001*
Mid sleep point, hour	3.82 (1.34)	4.02 [2.85, 4.67]	0.687

Abbreviations: BMI: Body Mass Index; Alcohol intake units per week (u/w): Alcohol units per week; Tobacco intake u/w: Tobacco (cigarettes) units per week; TALS-SR: Trauma and Loss Spectrum–Lifetime version; PTE: potentially traumatic events; PSQI: Pittsburgh Sleep Quality Index total score; SE: sleep efficiency, WASO: Wake After Sleep Onset; TST: total sleep time. Acrophase is reported as hours and minutes (hh:mm). TST and Mid sleep point are reported as fractions of hours. Results are presented in mean (SD: standard deviation) and median (IQR: Interquartile range). (*) Shapiro-Wilk normality test p-value < 0.05 = distribution of the data is significantly different from normal distribution.

Table S2. Post-hoc analyses p-values

	ET vs. NT	ET vs. MT	NT vs. MT
TALS-SR Reaction to Losses and PTE	0.072	0.080	0.861
TALS-SR Re-experiencing	0.009*	0.026*	0.637
TALS-SR Maladaptive coping	0.163	0.048*	0.408
TALS-SR Symptomatic domains	0.061*	0.105*	<0.999
PSQI Total score	<0.001*	0.006*	0.981
SE	0.050*	0.009*	0.503
WASO	0.039*	0.039*	0.550
SRI	0.141	0.005*	0.270
Acrophase	0.054	<0.001*	0.022*
Mesor	0.402	0.138	0.029*
Interdaily stability	0.152	0.025*	0.138
Relative amplitude	0.512	0.008*	0.008*
Mid sleep point	0.001*	<0.001	<0.001

Post-hoc pairwise Mann-Whitney-Wilcoxon rank-sum test results are reported as follows:
a Significant difference between Evening type and Neither type groups.
b Significant difference between Evening type and Morning type groups.
c Significant difference between Neither type and Morning type groups.
(*) Significant values were considered at p-value $\leqslant 0.05$.
NS: Comparisons that did not reach significance after holm correction

Table S3. Multicollinearity test

Variables	Tolerance	VIF
Age	0.9760157	1.024574
Sex	0.9650482	1.036218
PSQI	0.3403995	2.937724
Chronotype (ET vs NT)	0.1208217	8.276661
Chronotype (ET vs MT)	0.1245224	8.030682
PSQI * Chronotype (ET vs NT)	0.1629387	6.137277
PSQI * Chronotype (ET vs MT)	0.1601741	6.243206

VIF is higher than 10 or tolerance is lower than 0.1 = significant multicollinearity that needs to be corrected.

Table S4. Comparison between chronotypes regarding clinical variables

	Evening Type	Neither type	Morning Type	p-value
n	27	33	14	
BD type II (%)	22 (81.5)	23 (69.7)	12 (85.7)	0.386
Age disease onset	17.00 [14.50, 20.00]	23.50 [16.75, 30.00]	19.00 [16.25, 32.50]	0.034*
Manic episodes	0.00 [0.00, 0.00]	0.00 [0.00, 2.00]	0.00 [0.00, 0.00]	0.496
Depressive episodes	4.00 [3.00, 5.00]	3.00 [2.00, 4.00]	3.00 [2.00, 4.00]	0.179
Hypomanic episodes	2.00 [1.00, 3.50]	2.00 [1.00, 3.00]	3.00 [1.25, 4.00]	0.620
Admissions	0.00 [0.00, 1.00]	0.00 [0.00, 2.00]	0.50 [0.00, 1.75]	0.939
Number of suicides	0.00 [0.00, 1.00]	0.00 [0.00, 1.00]	0.00 [0.00, 0.00]	0.354
Antidepressants = YES (%)	20 (74.1)	24 (72.7)	10 (71.4)	0.983
Antidepressants SSRI = YES (%)	16 (59.3)	19 (57.6)	5 (35.7)	0.308
Antidepressant Triciclic = YES (%)	6 (22.2)	7 (21.2)	4 (28.6)	0.855
Antidepressant Other = YES (%)	3 (11.1)	5 (15.2)	3 (21.4)	0.677
Lithium = YES (%)	12 (44.4)	16 (48.5)	4 (28.6)	0.446
Other mood stabilizers = YES (%)	23 (85.2)	30 (90.9)	11 (78.6)	0.511
Benzodiazepines = YES (%)	5 (18.5)	3 (9.1)	3 (21.4)	0.442
Antipsychotics = YES (%)	16 (59.3)	15 (45.5)	10 (71.4)	0.230
Anxiolytics = YES (%)	25 (92.6)	28 (84.8)	13 (92.9)	0.559
Panic Disorder = YES (%)	21 (77.8)	21 (63.6)	9 (64.3)	0.459
PTSD = YES (%)	12 (44.4)	11 (33.3)	4 (28.6)	0.533
OCD = YES (%)	9 (33.3)	5 (15.2)	6 (42.9)	0.096
Eating Disorder = YES (%)	3 (11.1)	4 (12.1)	2 (14.3)	0.957
Substance Abuse = YES (%)	3 (11.1)	5 (15.2)	2 (14.3)	0.898

Abbreviations: PTSD: Post-traumatic stress disorder; OCD: Obsessive-Compulsive Disorder.

(*) Significant values were considered at *p*-value ≤ 0.05.
